# Supplementary material for: tBid‐Mediated Genetic Ablation of Connective Tissue Cells Reveals Their Key Regulatory Function During Limb Regeneration in Axolotls
Source: Adv Sci (Weinh). 2026 May 27;13(41):e24339. doi: 10.1002/advs.202524339 (PMC13335966; doi:10.1002/advs.202524339)
Supplement: Supplementary file 1 — Supporting File: advs75799‐sup‐0001‐SuppMat.docx [file ADVS-13-e24339-s001.docx]

Supporting Information

tBid Mediated Genetic Ablation of Connective Tissue Cells Reveal Their Key Regulatory Function During Limb Regeneration in Axolotls

**Yan Hu^1,2,#^, Weimin Feng^3,4,#^, Zitian He^1,2,#^, Jiayi Zeng^5,#^, Jingyi Tang^2^, Shulin Li^2^, Tongbo Yu^3^, Sulei Fu^1,2^, Hui Ma^6^, Binbin Lu^6^, Yanmei Liu^7,*^, Ji-Feng Fei^1,2,6,8,*^**

1. Guangdong Cardiovascular Institute, Guangdong Provincial People's Hospital, Guangdong Academy of Medical Sciences, Guangzhou, Guangdong 510080, China.

2. Department of Pathology, Guangdong Provincial People’s Hospital, Guangdong Academy of Medical Sciences, Southern Medical University, Guangzhou 510080, China.

3. Guangdong Provincial Key Laboratory of Medical Immunology and Molecular Diagnostics, The First Dongguan Affiliated Hospital, Guangdong Medical University, Dongguan, Guangdong, 523808, China.

4. Department of Biotechnology, School of Basic Medical Sciences, Guangdong Medical University, Dongguan, Guangdong, 523808, China.

5. Key Laboratory of Brain, Cognition and Education Science, Ministry of Education, China; Institute for Brain Research and Rehabilitation, and Guangdong Key Laboratory of Mental Health and Cognitive Science, South China Normal University, Guangzhou 510631, China.

6. School of Basic Medical Sciences, Southern Medical University, Guangzhou 510515, China.

7. Guangdong Engineering Research Center of Precision Detection and Modulation of Human Microbiome, School of Life Sciences, South China Normal University, Guangzhou 510631, China.

8. The Innovation Centre of Ministry of Education for Development and Diseases, School of Medicine, South China University of Technology, Guangzhou 510006, China.

# These authors contributed equally to this work.

* Corresponding authors.

E-mail addresses: [yanmeiliu@m.scnu.edu.cn](mailto:yanmeiliu@m.scnu.edu.cn) (Y. Liu), [jifengfei@gdph.org.cn](mailto:jifengfei@gdph.org.cn) (J-F. Fei)

Key words: axolotl, cell ablation, connective tissue, limb regeneration, Prrx1

Running title: Limb regeneration after CT ablation

**Supplementary Figures and Figure Legends**


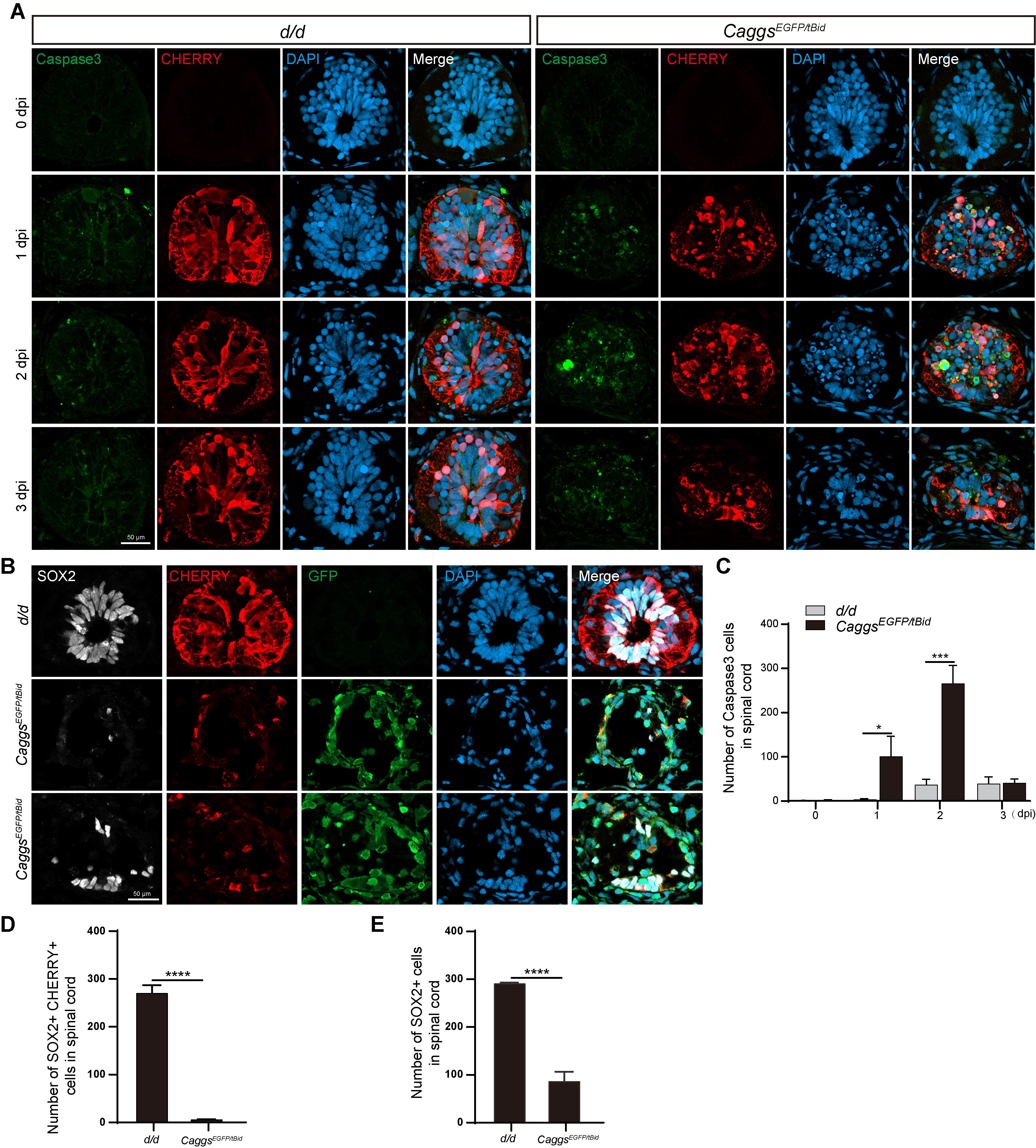


**Figure S1. Evaluation of the speed and efficiency of *tBid*-mediated cell ablation via electroporation in axolotls.**

A) Immunofluorescence staining for CHERRY (red), cleaved Caspase3 (green) with DAPI (blue) on tail cross-sections from control (*d/d,* left panels) and experimental group (*Caggs^EGFP/tBid^*, right panels) at 0, 1, 2 and 3 dpe. In experimental group, Caspase3 signals appeared at 1 dpe, peaked at 2 dpe, and declined by 3 dpe. B) Immunofluorescence staining for SOX2 (white), CHERRY (red), GFP (green) with DAPI (blue) on tail cross-sections from control (upper panels) and experimental group (middle and lower panels) at 4 dpe. SOX2+ were markedly reduced in the experimental group, with nearly complete elimination (>90%) within CHERRY-positive regions. C) Quantification of Caspase3-positive cells in spinal cord at 0, 1, 2, and 3 dpe in control (gray bars, n = 3) and experimental (black bars, n = 3) groups. D) and E) Quantification of double-positive (SOX2+/CHERRY+) cells (D) and SOX2+ cells (E) in spinal cord at 4 dpe (n = 3, each). Data were analyzed by unpaired two-tailed Student’s t-test and represented as mean ± SEM, *p<0.05, ***p<0.001, ****p<0.0001. Scale bar: 50 μm.


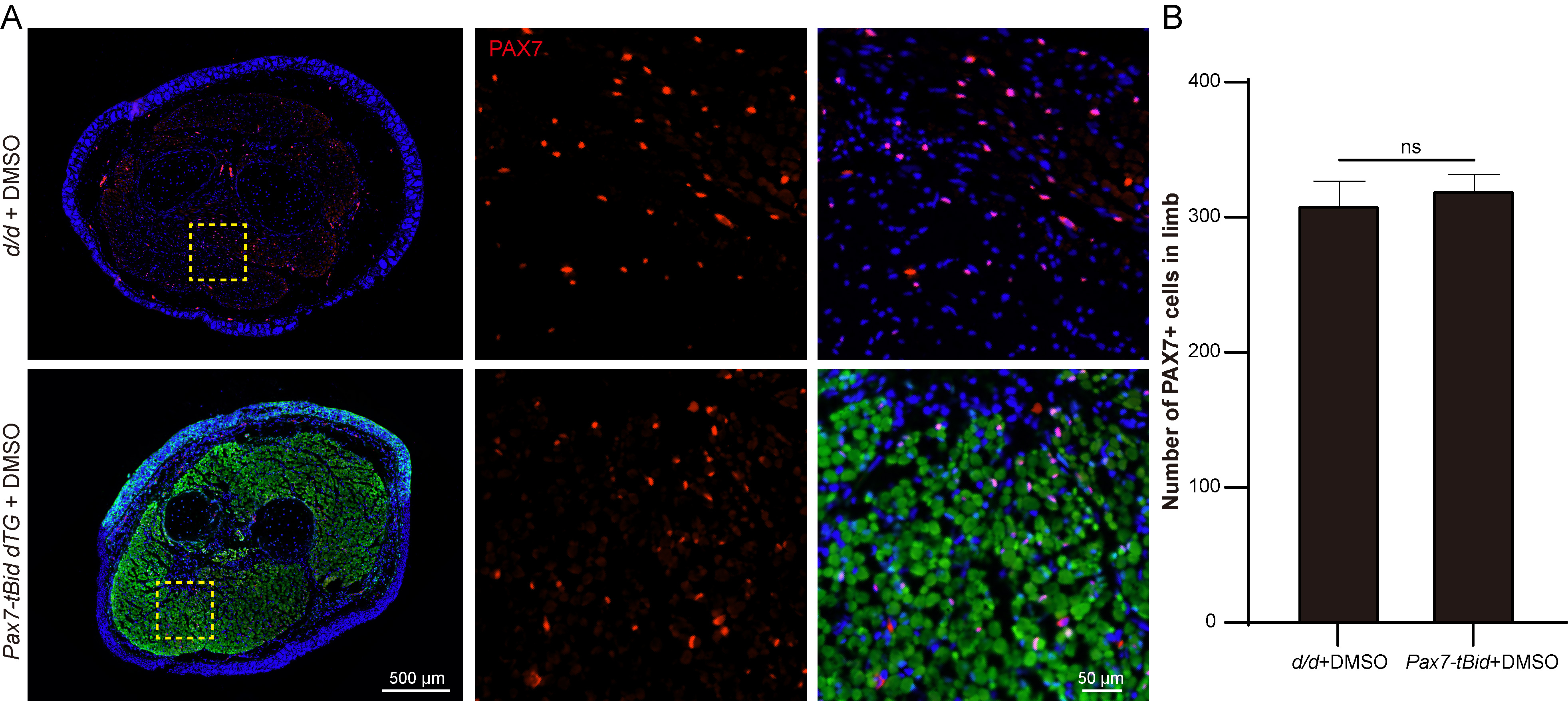


**Figure S2. DMSO treatment does not induce PAX7+ cell ablation in transplanted *Pax7-tBid dTG* limb.**

A) Immunofluorescence staining for PAX7 (red) and endogenous EGFP fluorescence (green) with DAPI (blue) on limb cross-sections from *d/d* (upper panels) and *Pax7-tBid dTG* transplanted limb (lower panels) at 8 days post-treatment (dpt). PAX7 signal is no significant difference between the transplanted *Pax7-tBid dTG* and *d/d* limb. B) Quantification of PAX7+ cells in limb tissues at 8dpt (n=3 each). Data were analyzed by unpaired two-tailed Student’s t-test and represented as mean ± SEM, ns, not significant. Scale bars: 500 μm in (A) left; 50 μm in (A) right.


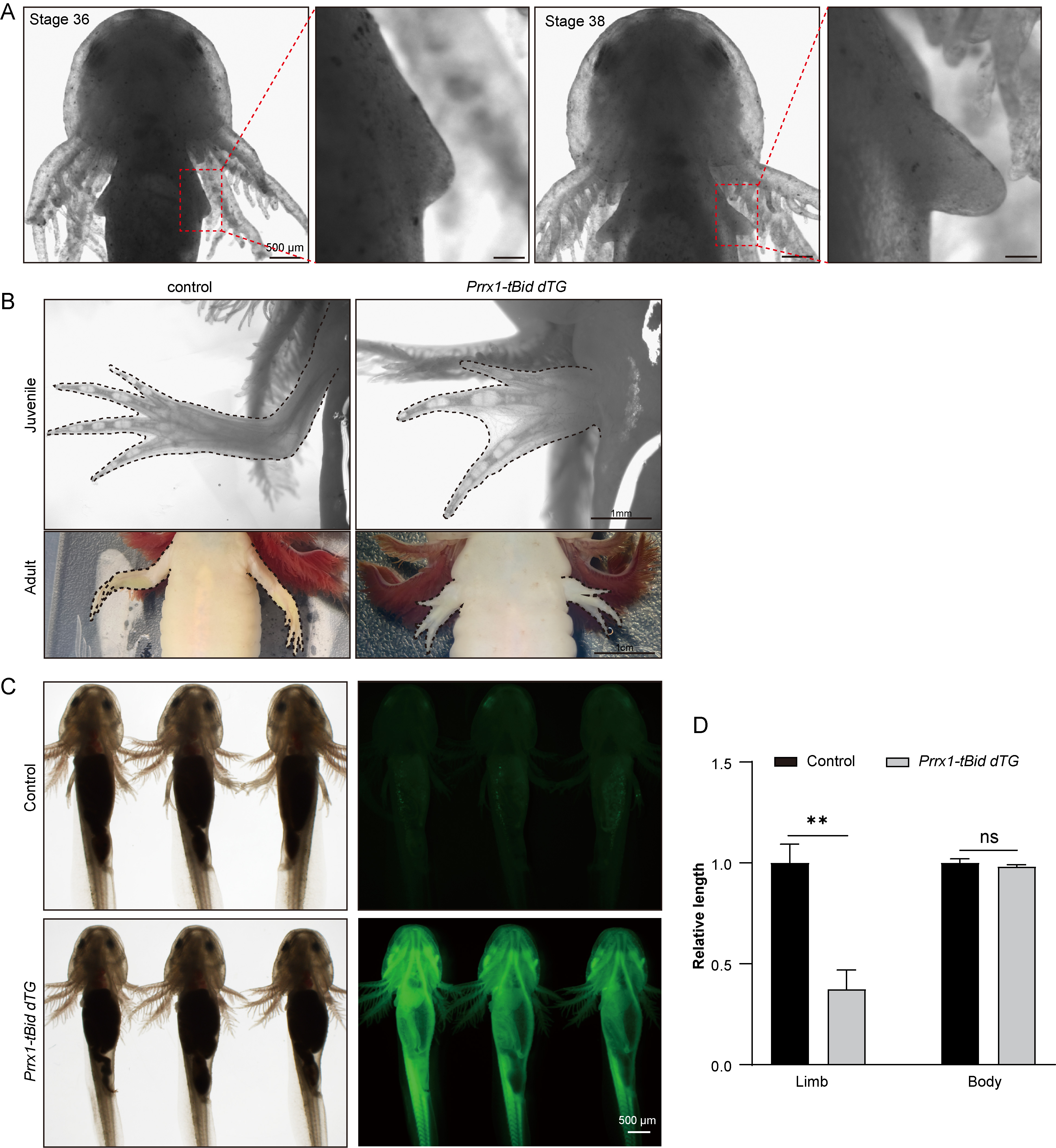


**Figure S3. Phenotype characterization after 4-OHT treatment in *Prrx1-tBid dTG* axolotls*.***

A) Representative live image of a *Prrx1-tBid dTG* limb bud prior to 4-OHT treatment during early development. The right panels show higher-magnification views of the regions outlined by red dashed boxes. B) Long-term observation of representative limbs from early 4-OHT treated control (*Caggs^EGFP/tBid^*; left panels) and *Prrx1-tBid dTG* (right panels) axolotls at juvenile and adult stages. In the most severe class of CT ablation-induced defects, digits formed in the complete absence of the stylopod, zeugopod, and autopod in *Prrx1-tBid dTG* axolotls. These defects remained stable throughout subsequent development, with no evidence of catch-up growth of the missing proximal segments (stylopod, zeugopod, or autopod) at the juvenile and adult stage. C) Live imaging of Bright-field (left) and EGFP fluorescence (right) of control (*Prrx1^Cre-ERT^,* upper panel) and *Prrx1-tBid dTG* (lower panel) axolotls at 21 days post-treatment (dpt). The limb of *Prrx1-tBid dTG* axolotl is notably smaller than that of the *Prrx1^Cre-ERT^*. D) Quantification of relative limb length and body length in control (black bars, n =5) and *Prrx1-tBid dTG* (gray bars, n =5) axolotls. Limb length was measured from the limb stump to the distal-most tip; body length was measured from snout to tail tip. Data were analyzed by unpaired two-tailed Student’s t-test and represented as mean ± SEM, ***p* <0.01; n.s., not significant. Scale bars: 1 mm (juvenile), 1 cm (adult), 500 μm.


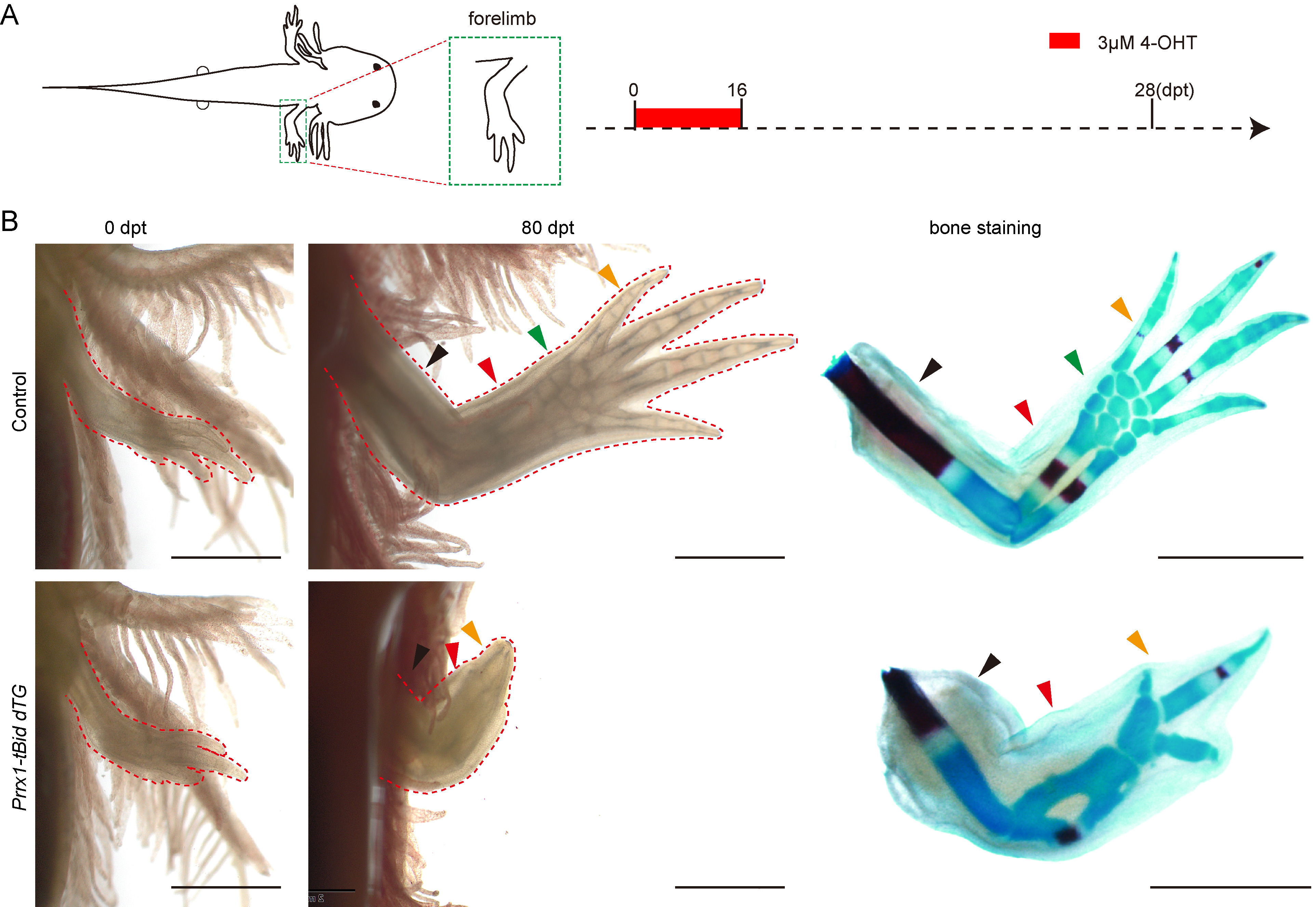


**Figure S4. Late ablation of CT cells causes forelimb defects during development.**

A) Timeline of 4-OHT administration during late forelimb development in *Prrx1-tBid dTG*. B) Representative images of development limb of control (*Caggs^EGFP/tBid^*, upper panel) and *Prrx1-tBid dTG* (lower panel) axolotls at 0 and 80 dpt. The right column shows the skeletal elements of control (*Caggs^EGFP/tBid^*, upper panel) and *Prrx1-tBid dTG* (lower panel) via alcian blue/alizarin skeletal staining. Red dashed lines outline the shape of limb. Black arrowheads indicate the stylopods; red arrowheads indicate the zeugopods; green arrowheads indicate the autopods; yellow arrowheads indicate the digits. Scale bars: 1 mm.


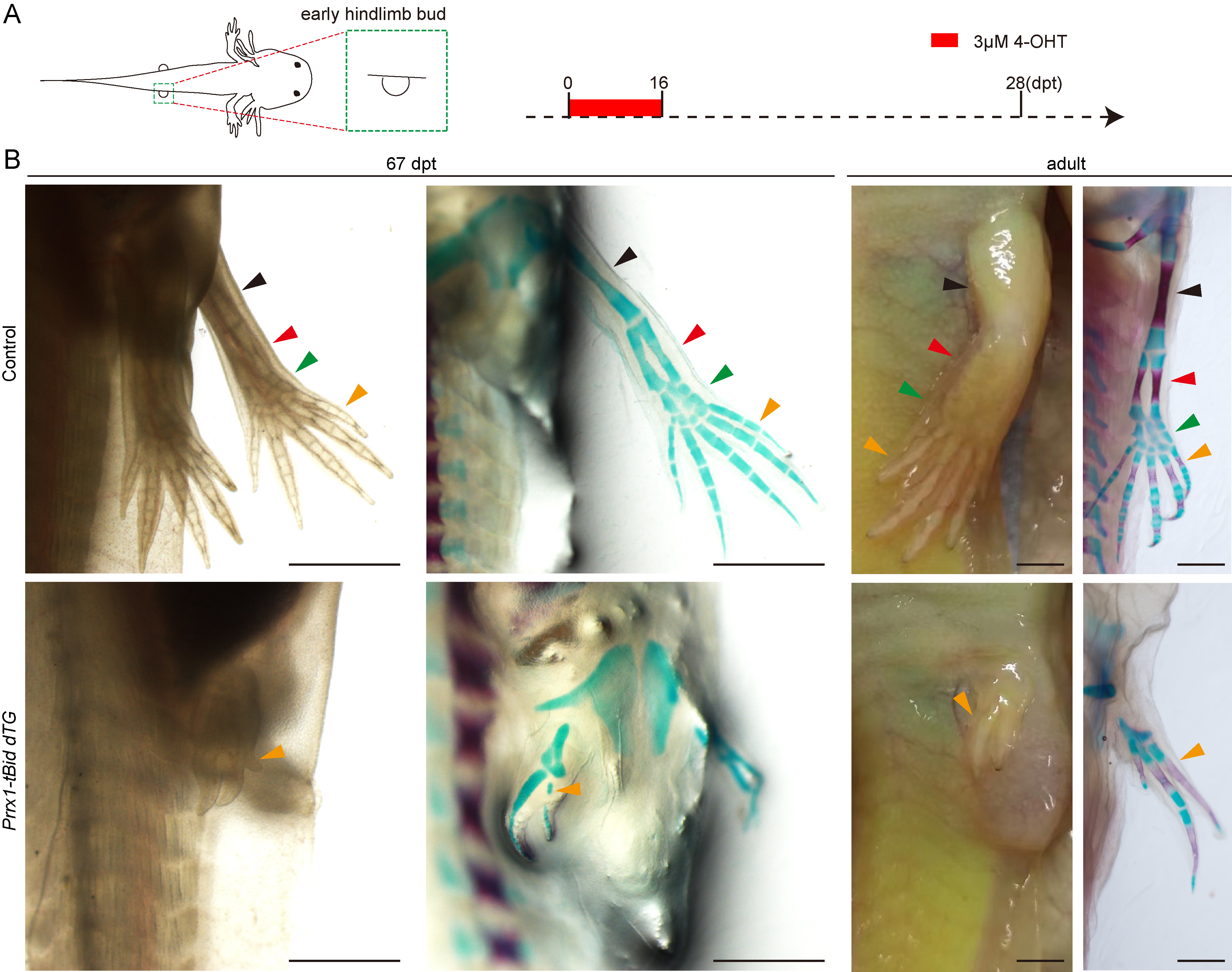


**Figure S5. CT cell ablation cause hindlimb defects during development.**

A) Timeline of 4-OHT administration during early hindlimb development in *Prrx1-tBid dTG*. B) The representative images of hindlimb development in control (*Caggs^EGFP/tBid^*, upper panel) and *Prrx1-tBid dTG* (lower panel) axolotls, with corresponding alcian blue/alizarin red skeletal staining, at 67dpt (left) and adulthood (right). Black arrowheads indicate the stylopods; red arrowheads indicate the zeugopods; green arrowheads indicate the autopods; yellow arrowheads indicate the digits. Scale bars: 1 mm.


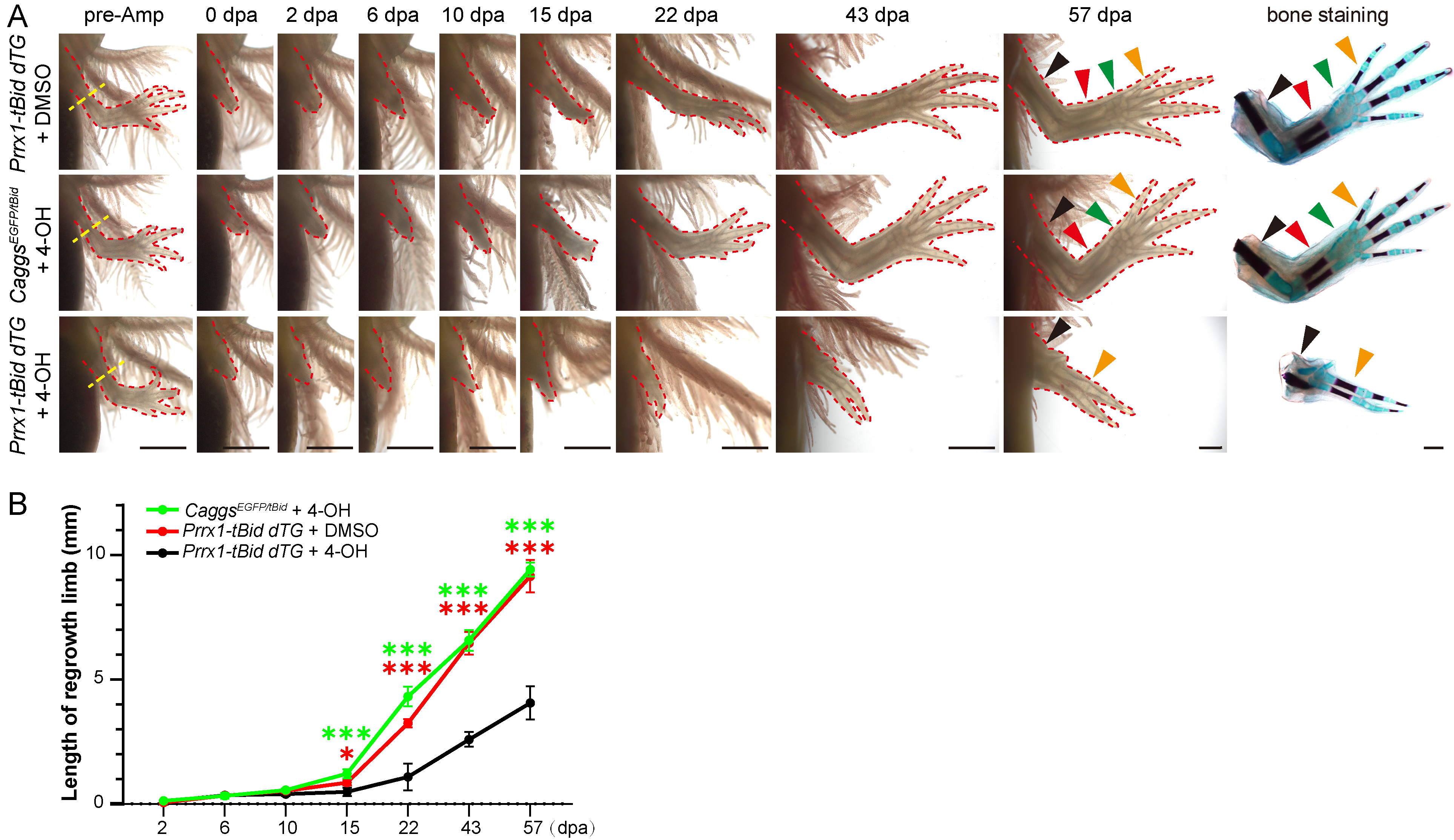


**Figure S6. CT cells are crucial for proper limb regeneration in axolotls.**

A) Representative time-series images of limb regeneration from all groups, with corresponding Alcian blue/Alizarin red skeletal staining at 57 days post-amputation (dpa). Limb regeneration defected in the *Prrx1-tBid dTG* group treated with 4-OHT (lower panel; the same animal as in Figure 3B) relative to both the DMSO-treated *Prrx1-tBid dTG* (upper panel) and the 4-OHT-treated groups (middle panel; the same animal as in Figure 3B). Yellow dashed lines indicate amputation planes. Red dashed lines outline the shape of limb. Black arrowheads indicate the stylopods; red arrowheads indicate the zeugopods; green arrowheads indicate the autopods; yellow arrowheads indicate the digits. B) Quantification of regenerated limb length over time (n = 3, each). Limb length was measured from the amputation plane (stump) to the most distal tip of the regenerate. For quantification, data were analyzed by unpaired two-tailed Student’s t-test and represented as mean ± SEM, **p* <0.05; ****p* <0.001. Scale bars: 1 mm.


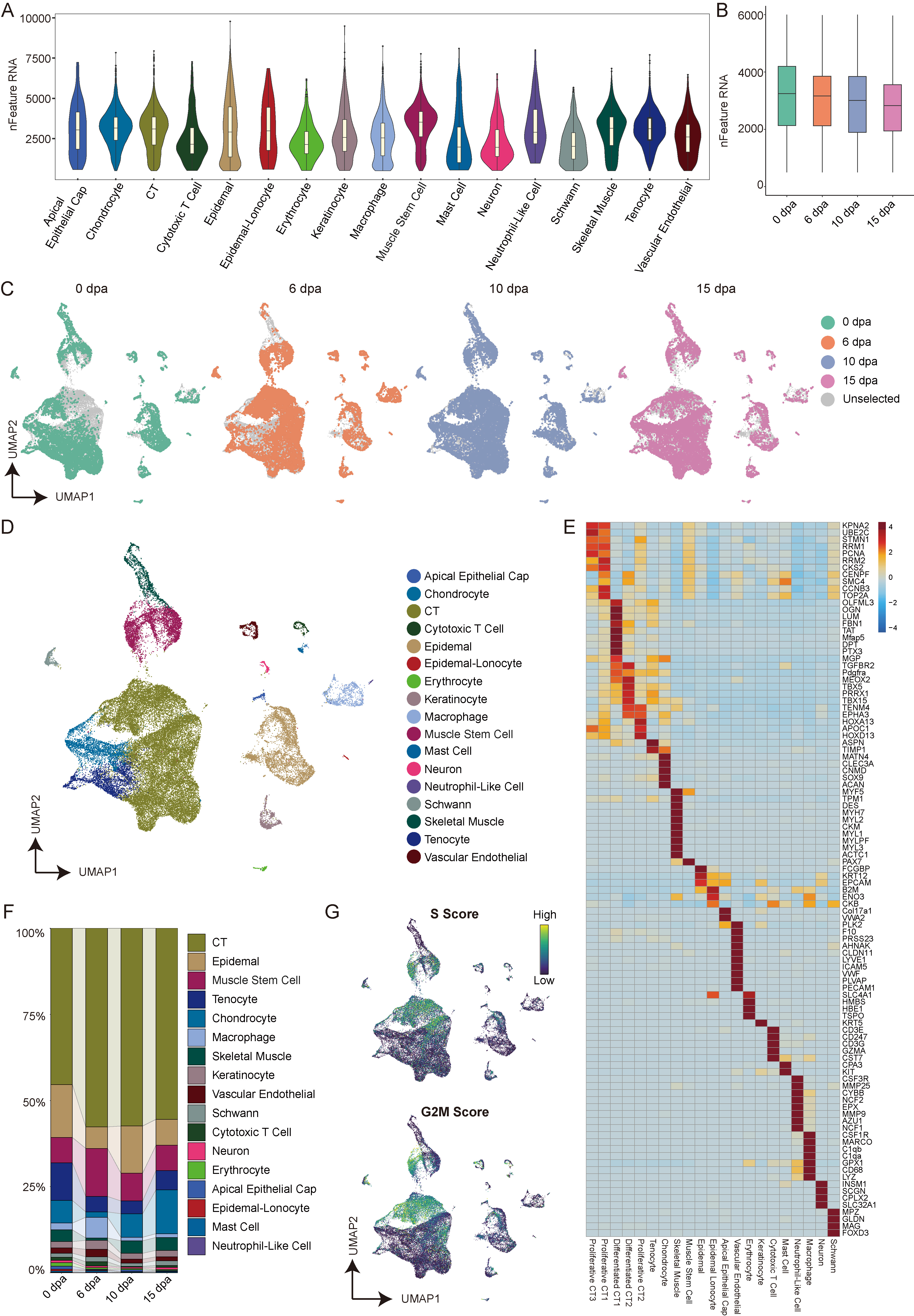


**Figure S7. Single-cell transcriptomic profiling of limb regeneration in control axolotls.**

A) Violin plot illustrating the number of genes distribution for each cell type. B) Violin plot illustrating the number of genes distribution for each library. C) The UMAP visualization represents cells from the four stages in control (*Caggs^EGFP/tBid^*) limbs regeneration, with colors corresponding to each stage. D) A UMAP visualization 17 cell types representing the four stages in control limbs regeneration. E) Heatmap showing the mean expression of marker genes of 21 cell types shown in Figure 5A. F) Stacked bar plots display the cellular composition of each sample, represented as the relative abundance of each cell type. G) UMAP visualization depicts cell cycle gene module activity, with S scores (left) and G2M scores (right).


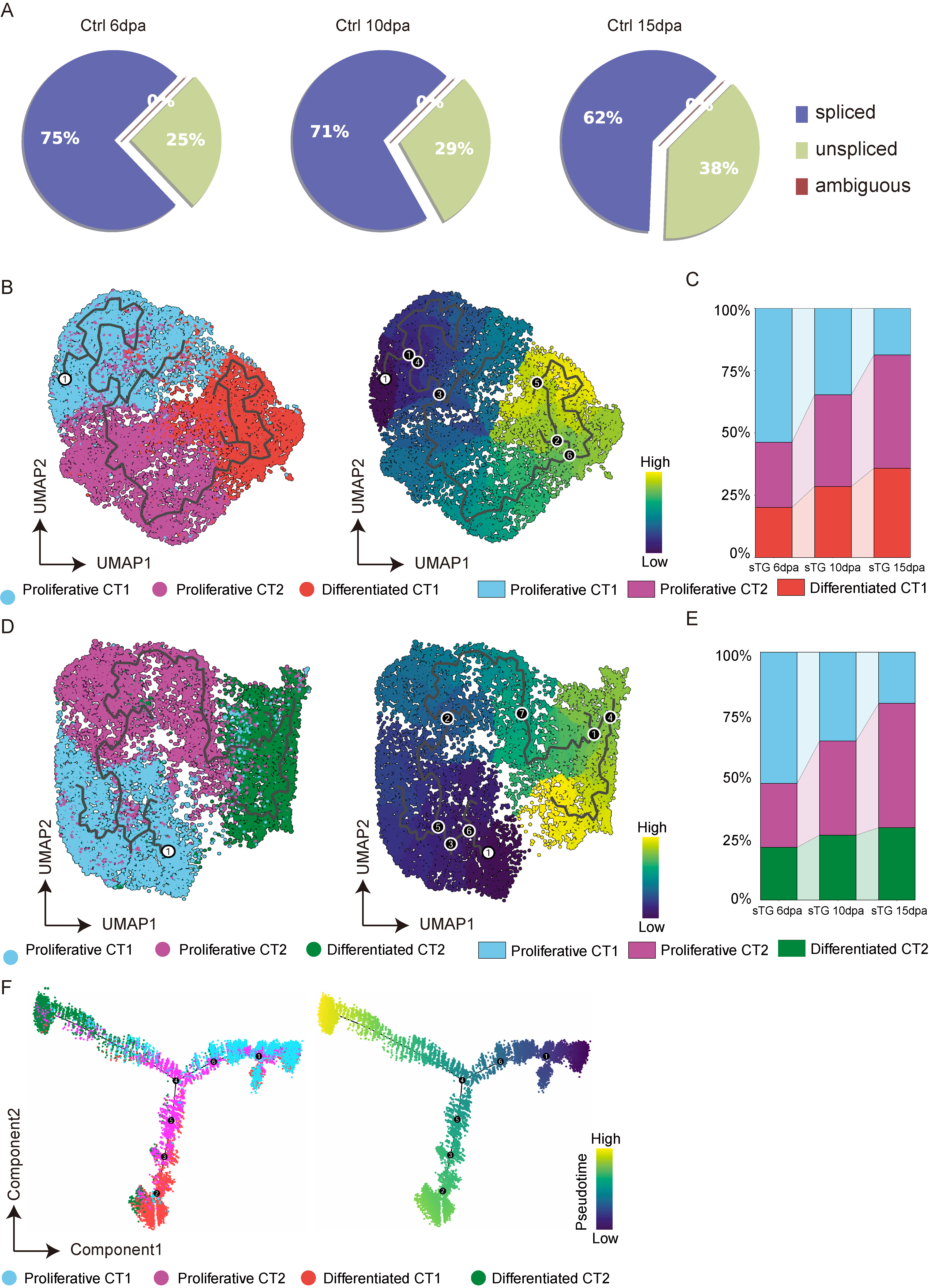


**Figure S8. scRNA-seq reveals the regenerative lineage of CT subpopulations in the control group.**

A) Pie chart showing the proportions of spliced, unspliced, and ambiguous matrices in the scRNA data of the control group. Note: Ctrl, Control (*Caggs^EGFP/tBid^*). B) and D) Monocle3 analysis of the differentiation trajectories of CT subpopulations in the control group. Of these, (B) UMAP plot illustrating the lineage transition among proliferative CT1, proliferative CT2, and differentiated CT1; (D) UMAP plot showing the lineage transition among proliferative CT1, proliferative CT2, and differentiated CT2. C) and E) Bar plots showing the proportions of relevant cell types within the lineages presented in (B) and (D), respectively. F) Monocle2 reveals two distinct differentiation paths of CT subpopulations in the controls.


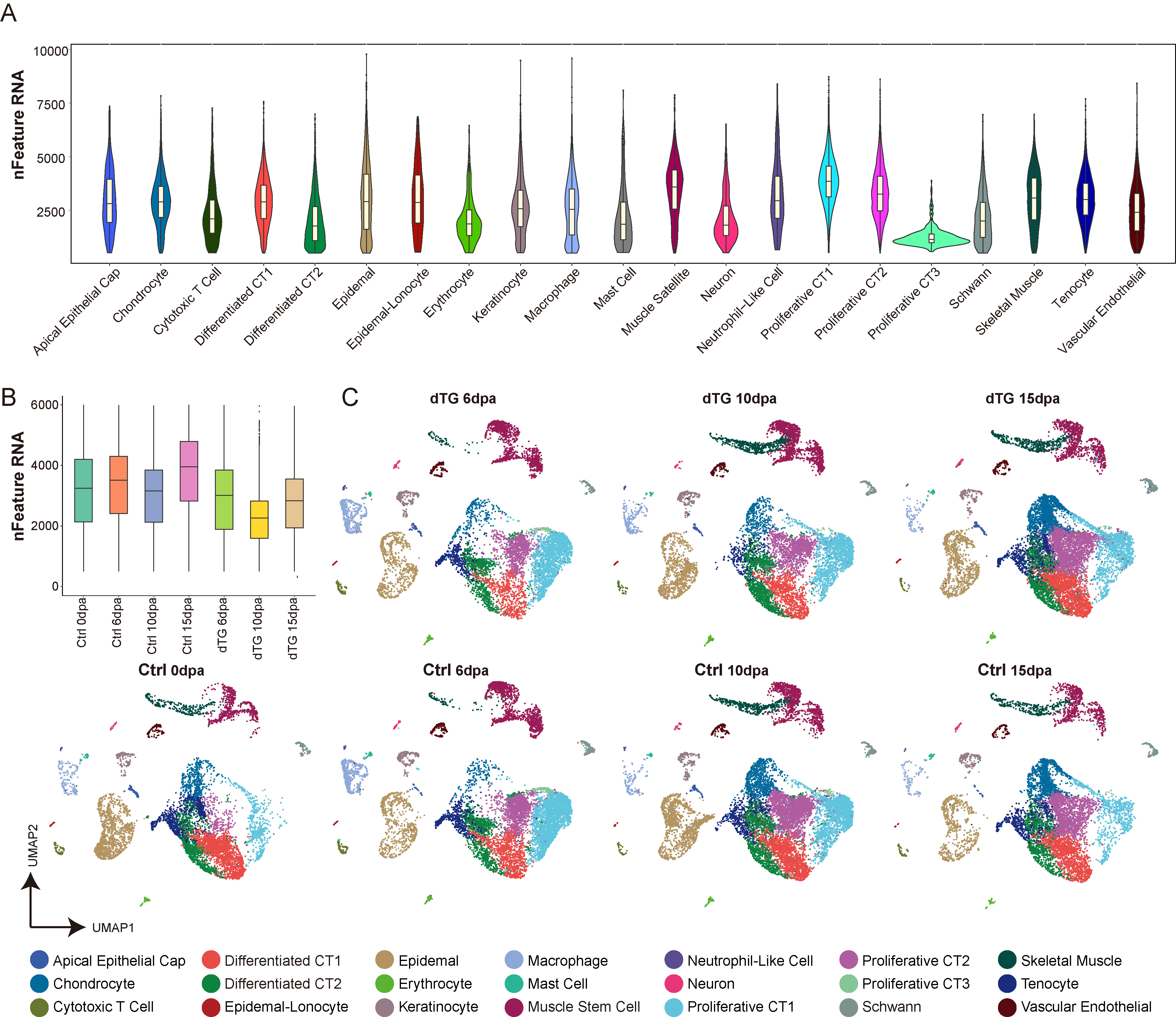


**Figure S9. Quality control for scRNA-seq of *Prrx1-tBid dTG* and control limbs.**

A) Violin plot illustrating the number of genes distribution for each cell type in *Prrx1-tBid dTG* and control (Ctrl, *Caggs^EGFP/tBid^*) limbs regeneration. B) Violin plot illustrating the number of genes distribution for each library in *Prrx1-tBid dTG* and control limbs regeneration. C) UMAP visualization 21 cell types representing the seven stages in *Prrx1-tBid dTG* and controls limbs regeneration.

**
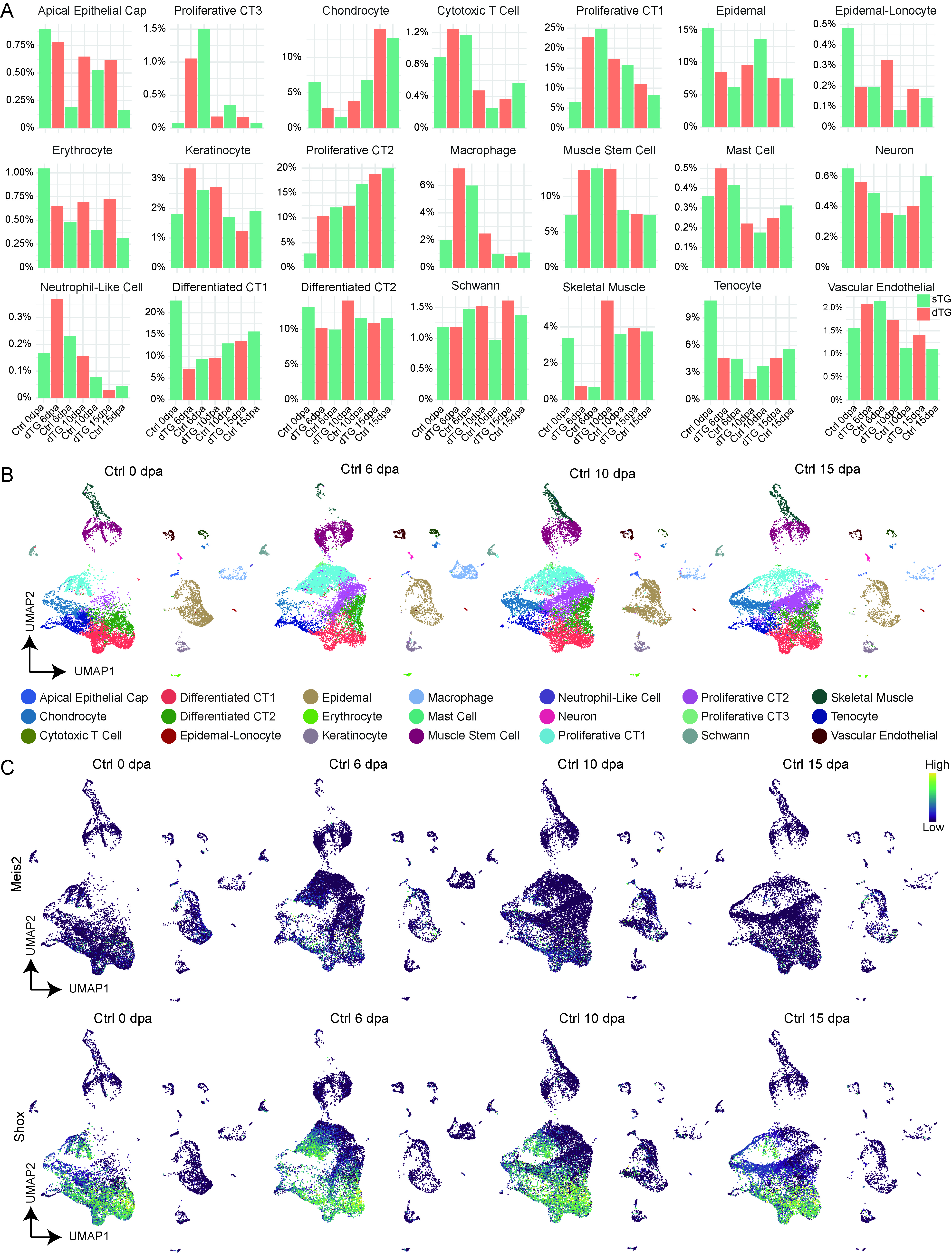
**

**Figure S10. Limb regeneration cell dynamics and proximal** **marker expression in *Prrx1-tBid dTG* and control axolotls.**

A) Bar plots depict stage-specific cell type proportions relative to total cells per stage during limb regeneration in *Prrx1-tBid dTG* and control (Ctrl, *Caggs^EGFP/tBid^*) axolotls. B) UMAP plot of cell types from four regenerative stages in controls, colored by cell type. C) Expression of proximal markers *Meis2* and *Shox* on the UMAP across four regenerative stages in controls.
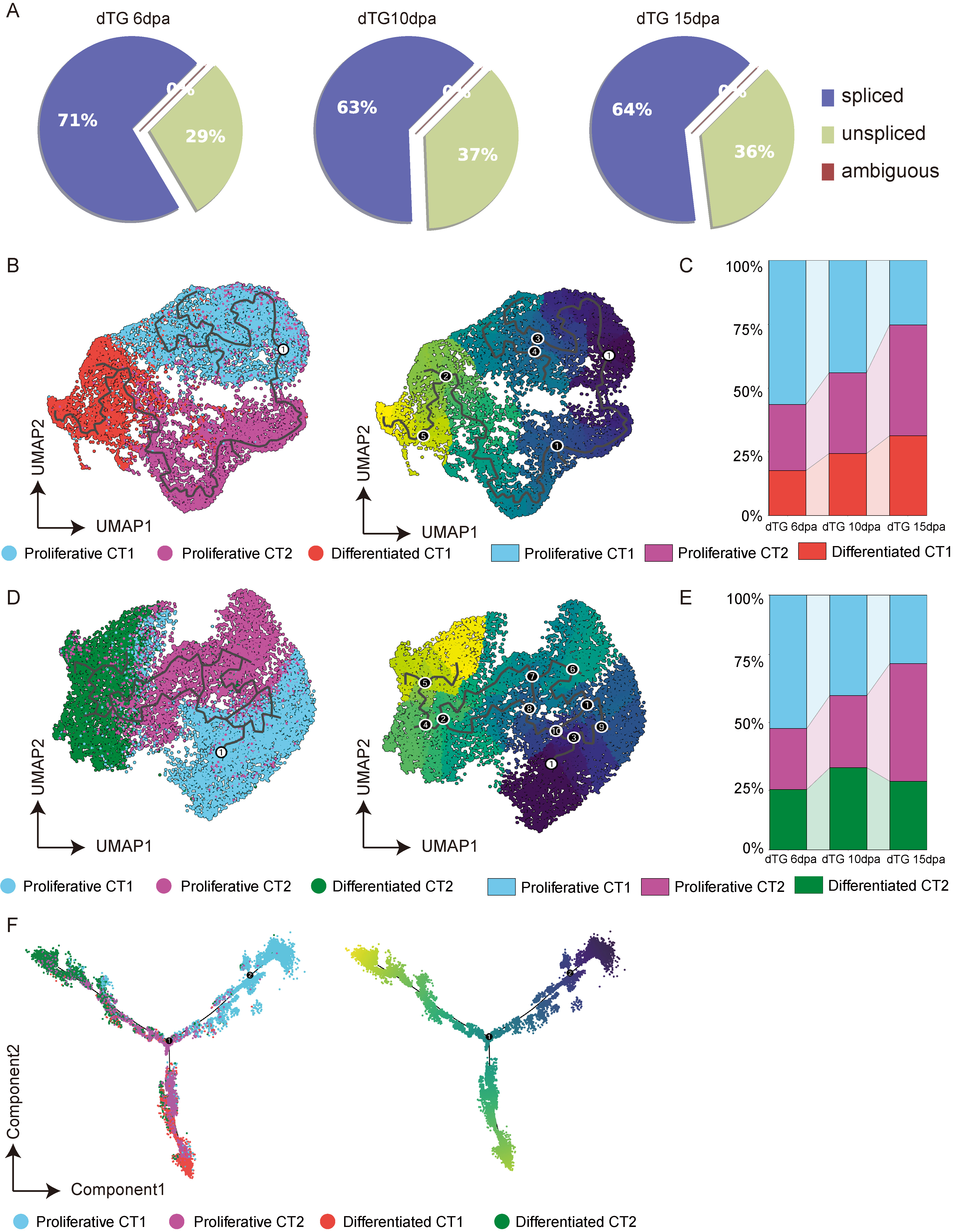


**Figure S11. Single-cell RNA sequencing reveals the regenerative differentiation trajectory of CT subpopulations in *Prrx1-tBid dTG* axolotls.**

A) Pie chart showing the proportions of spliced, unspliced, and ambiguous reads in the single-cell transcriptomic data derived from *Prrx1-tBid dTG* axolotls (dTG). B, D) Monocle3 analysis of the differentiation trajectories of CT subpopulations in the *Prrx1-tBid dTG* axolotl. In (B), the UMAP plot depicts lineage progression among proliferative CT1, proliferative CT2, and differentiated CT1 populations. In (D), the UMAP plot shows lineage relationships among proliferative CT1, proliferative CT2, and differentiated CT2 populations.C, E) Bar plots showing the relative proportions of each cell type along the differentiation trajectories corresponding to panels (B) and (D), respectively. F) Monocle2 analysis identifies two principal differentiation trajectories of CT subpopulations in *Prrx1-tBid dTG* axolotls.

**
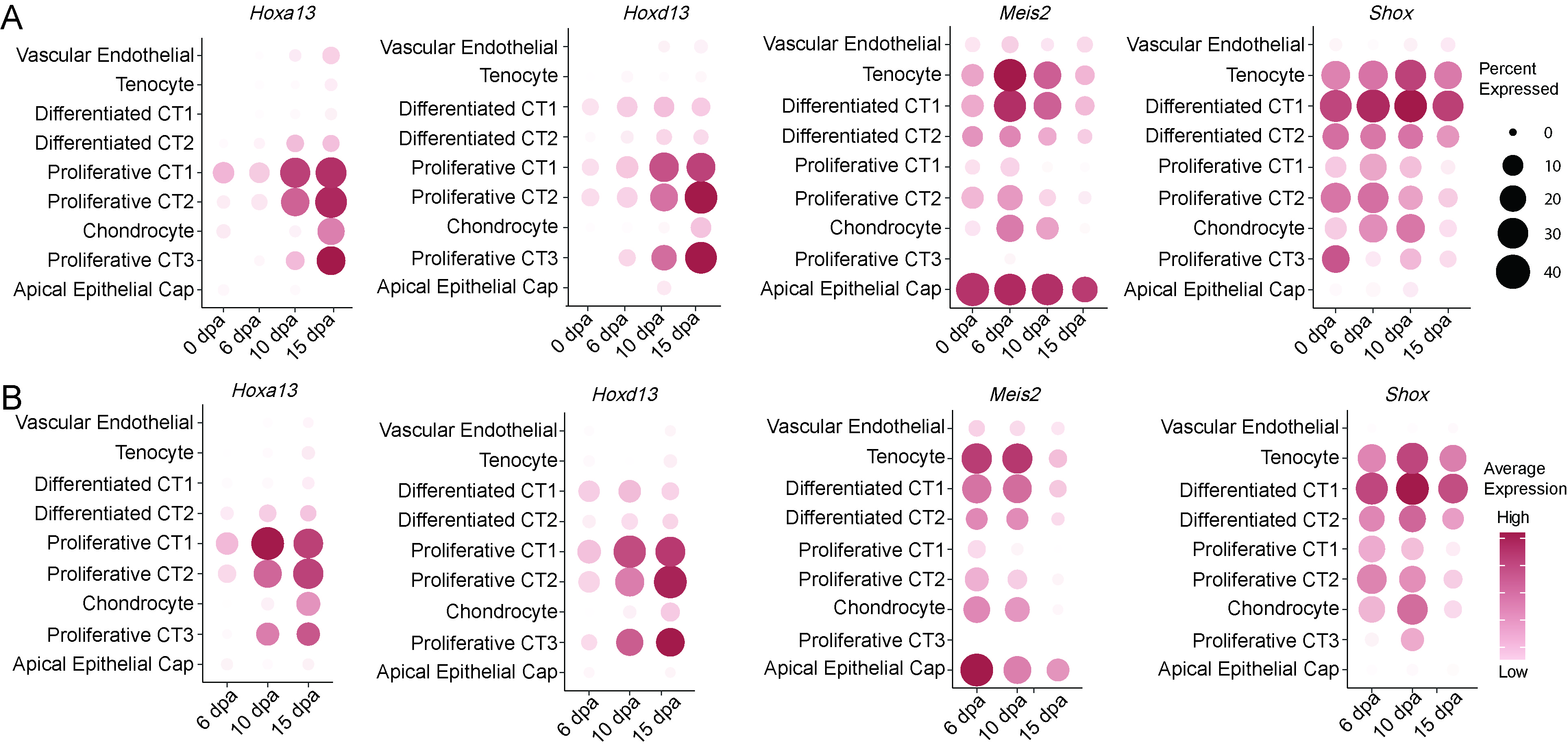
Figure S12. Distal and proximal gene expression patterns between control and *Prrx1-tBid dTG* axolotls**

A) and B) Dotplot showing the distal (*Hoxa13, Hoxd13*) and proximal (*Meis2, Shox*) marker expression during limb regeneration in the control (*Caggs^EGFP/tBid^*) (A) and *Prrx1-tBid dTG* (B) axolotls.


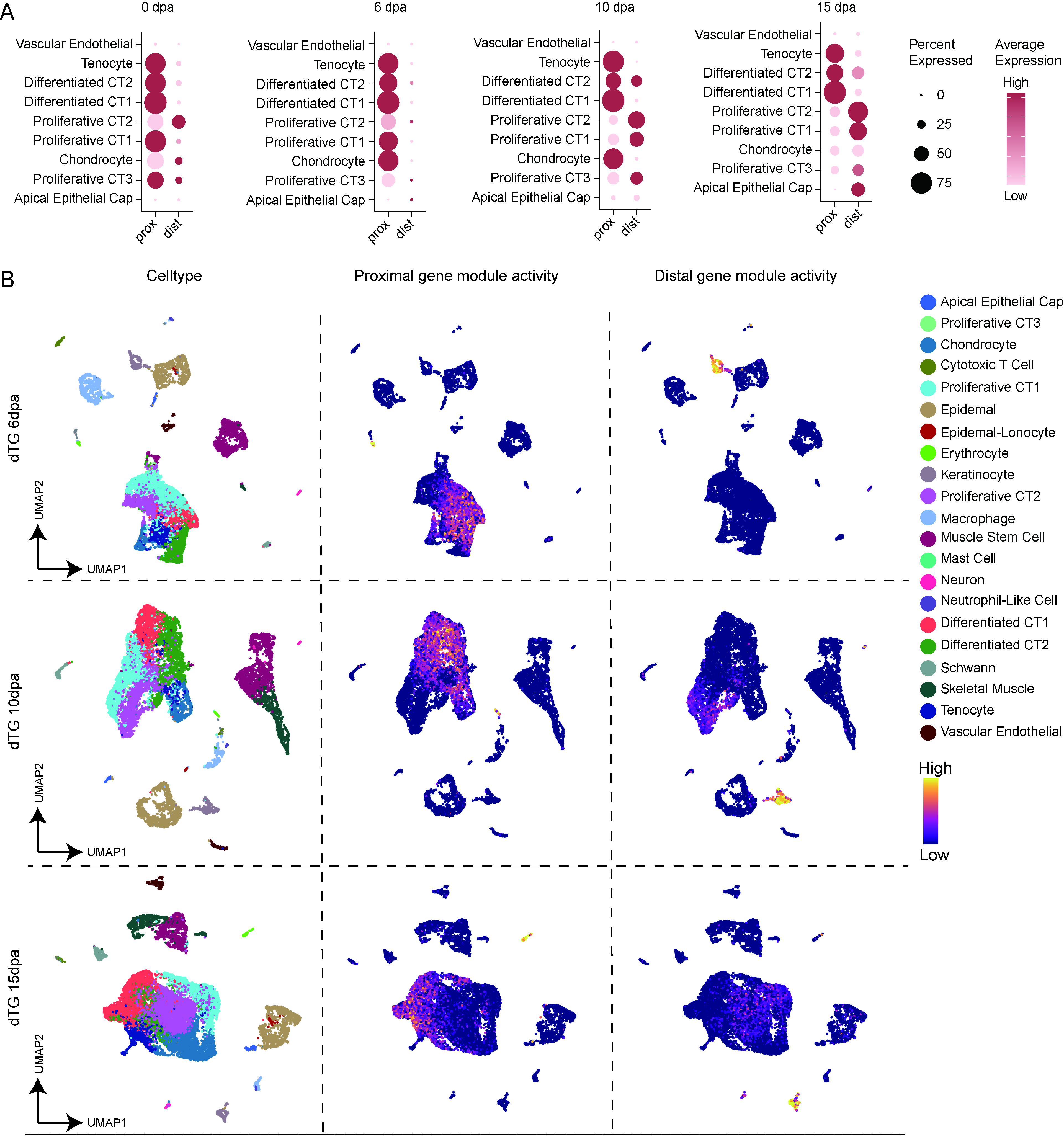


**Figure S13 Comparison and distribution of gene module activity in regenerating axolotl limbs.**

A) The dotplot compares gene module activity enrichment between distal (dist) and proximal (prox) regions during limb regeneration in control (*Caggs^EGFP/tBid^*) axolotls at 0, 6, 10, and 15 dpa. B) UMAP display showing the enrichment distribution of distal and proximal gene module activity in *Prrx1-tBid dTG* axolotls at 6, 10, and 15 dpa.


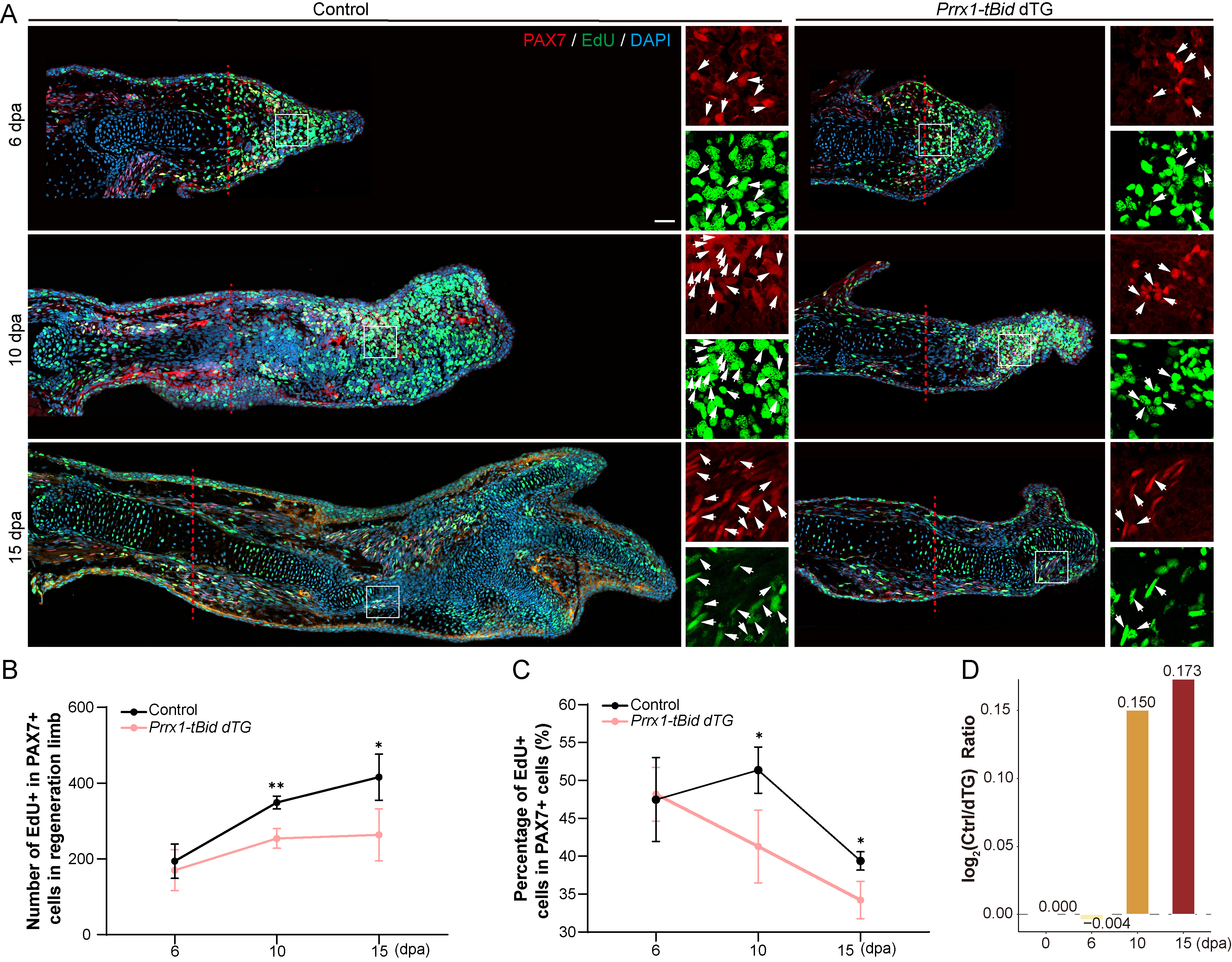


**Figure S14. CT cells ablation impairs muscle stem cell proliferation.**

A) Immunofluorescence images of PAX7 (red), EdU (green), and DAPI (blue) on limb longitudinal-sections from control (*Caggs^EGFP/tBid^*) (left) and *Prrx1-tBid dTG* (right) axolotls at 6, 10 and 15 dpa. Red dashed lines indicate amputation planes. White boxes outline the higher magnification area of blastema, showing single-channel. White arrowheads indicate the EdU+ PAX7+ cells. B) and C) Quantification of the absolute number (B) and the ratio (C) of EdU+ cells among the PAX7+ cell population within the newly formed blastema and a combined 500 μm zone proximal to the amputation plane at 6, 10, and 15dpa in control (*Caggs^EGFP/tBid^*, black line, n = 3) and *Prrx1-tBid dTG* (red line, n = 3) axolotls. D) Bar chart showing the log2-transformed cell proportion ratios (control vs. *Prrx1-tBid dTG*) across all stages of scRNA-seq data. A ratio > 0 indicates enrichment in control, while a ratio < 0 indicates enrichment in *Prrx1-tBid dTG*. Scale bar: 100 μm. For quantification, data were analyzed by unpaired two-tailed Student’s t-test and represented as mean ± SEM, **p*<0.05; ***p*<0.01.


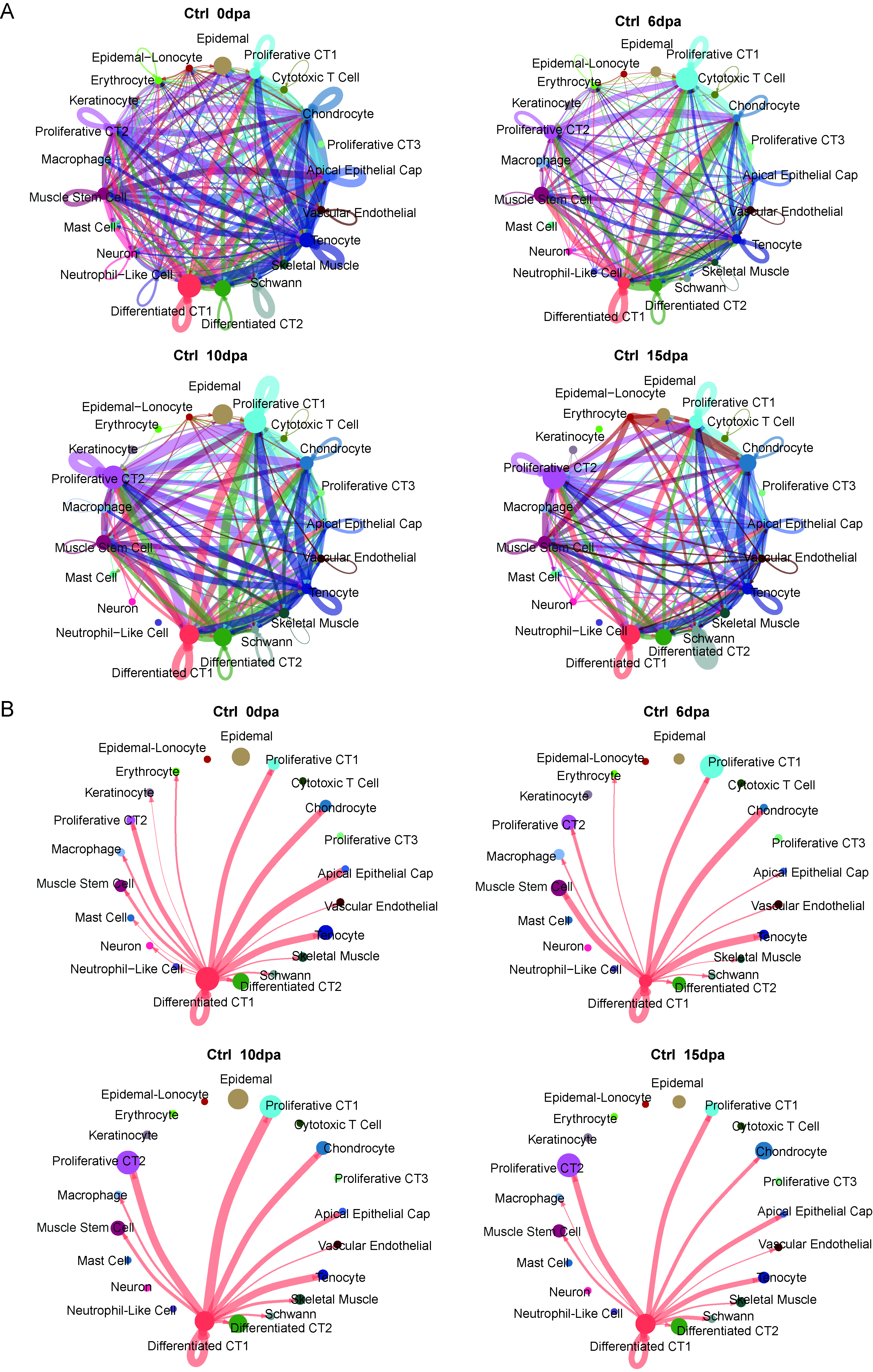


**Figure S15. scRNA-seq analysis of cell-cell communication networks in controls*.***

A) Chord diagram depicting cell type communication networks in controls (*Caggs^EGFP/tBid^*) across four regeneration stages (0, 6, 10 and 15 dpa). B) cell-cell communication between differentiated CT1 and other cell types in control across four regeneration stages.


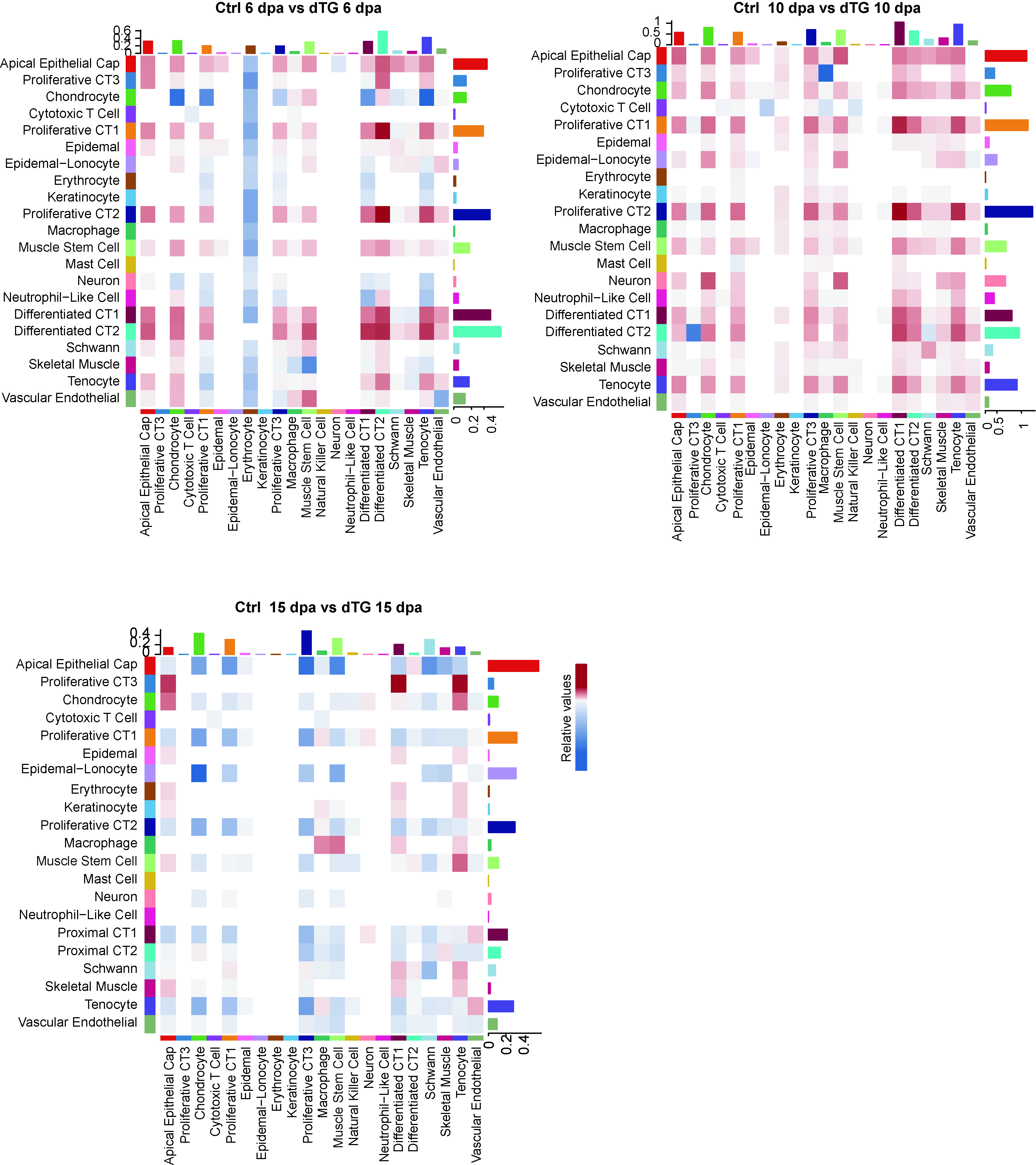


**Figure S16. Heatmap diagrams compare cell-cell interaction networks between *Prrx1-tBid dTG* and control (*Caggs^EGFP/tBid^*) at 6, 10, and 15 dpa.** Red indicates stronger signaling in *Prrx1-tBid dTG* axolotls relative to controls, whereas blue denotes weaker signaling in *Prrx1-tBid dTG* axolotls versus controls.**Supporting Information Table 1.**

Genes used for defining distal-proximal cells.

| Proximal_genes | | Distal_genes | |
| --- | --- | --- | --- |
| Gene ID | **Gene name** | **Gene ID** | **Gene name** |
| AMEX60DDU001036023 | *LOC106705246:2* | AMEX60DDU001026933 | *AMEX60DDU001026933* |
| AMEX60DD015653 | *AMEX60DD015653* | AMEX60DDU001013614 | *AMEX60DDU001013614* |
| AMEX60DDU001033785 | *AMEX60DDU001033785* | AMEX60DD027603 | *AMEX60DD027603* |
| AMEX60DD044027 | *VMD2:89* | AMEX60DDU001003497 | *AMEX60DDU001003497* |
| AMEX60DDU001002251 | *AMEX60DDU001002251* | AMEX60DDU001042089 | *AMEX60DDU001042089* |
| AMEX60DDU001005147 | *AMEX60DDU001005147* | AMEX60DDU001039046 | *AMEX60DDU001039046* |
| AMEX60DDU001029531 | *AMEX60DDU001029531* | AMEX60DDU001009762 | *HARBI1:107* |
| AMEX60DDU001020680 | *AMEX60DDU001020680* | AMEX60DDU001015843 | *AMEX60DDU001015843* |
| AMEX60DDU001035657 | *AMEX60DDU001035657* | AMEX60DDU001038474 | *ENV:229* |
| AMEX60DDU001014398 | *AMEX60DDU001014398* | AMEX60DDU001018784 | *AMEX60DDU001018784* |
| AMEX60DD053231 | *AMEX60DD053231* | AMEX60DD031109 | *AMEX60DD031109* |
| AMEX60DDU001014634 | *AMEX60DDU001014634* | AMEX60DD010431 | *TRIM39:32* |
| AMEX60DDU001008796 | *AMEX60DDU001008796* | AMEX60DDU001037306 | *AMEX60DDU001037306* |
| AMEX60DDU001026207 | *AMEX60DDU001026207* | AMEX60DD020038 | *AMEX60DD020038* |
| AMEX60DDU001041233 | *AMEX60DDU001041233* | AMEX60DDU001010125 | *AMEX60DDU001010125* |
| AMEX60DDU001000909 | *AMEX60DDU001000909* | AMEX60DDU001023235 | *AMEX60DDU001023235* |
| AMEX60DDU001026067 | *AMEX60DDU001026067* | AMEX60DDU001036829 | *AMEX60DDU001036829* |
| AMEX60DDU001017898 | *AMEX60DDU001017898* | AMEX60DDU001014526 | *AMEX60DDU001014526* |
| AMEX60DDU001020421 | *AMEX60DDU001020421* | AMEX60DDU001024167 | *AMEX60DDU001024167* |
| AMEX60DDU001021301 | *AMEX60DDU001021301* | AMEX60DD010887 | *TAP2* |
| AMEX60DDU001005842 | *AMEX60DDU001005842* | AMEX60DDU001017751 | *AMEX60DDU001017751* |
| AMEX60DDU001015402 | *AMEX60DDU001015402* | AMEX60DDU001027642 | *AMEX60DDU001027642* |
| AMEX60DDU001034785 | *AMEX60DDU001034785* | AMEX60DDU001040864 | *AMEX60DDU001040864* |
| AMEX60DDU001035434 | *AMEX60DDU001035434* | AMEX60DDU001038725 | *AMEX60DDU001038725* |
| AMEX60DDU001032541 | *AMEX60DDU001032541* | AMEX60DDU001020247 | *AMEX60DDU001020247* |
| AMEX60DD013447 | *AMEX60DD013447* | AMEX60DDU001014129 | *AMEX60DDU001014129* |
| AMEX60DD039927 | *LOC115089817:8* | AMEX60DDU001036305 | *AMEX60DDU001036305* |
| AMEX60DDU001031829 | *AMEX60DDU001031829* | AMEX60DD040981 | *AMEX60DD040981* |
| AMEX60DDU001013699 | *AMEX60DDU001013699* | AMEX60DDU001028376 | *AMEX60DDU001028376* |
| AMEX60DD007717 | *AMEX60DD007717* | AMEX60DDU001004023 | *AMEX60DDU001004023* |
| AMEX60DDU001018605 | *AMEX60DDU001018605* | AMEX60DDU001012986 | *LOC106733090:46* |
| AMEX60DDU001026790 | *AMEX60DDU001026790* | AMEX60DDU001013820 | *AMEX60DDU001013820* |
| AMEX60DDU001018776 | *AMEX60DDU001018776* | AMEX60DD052922 | *AMEX60DD052922* |
| AMEX60DDU001030638 | *AMEX60DDU001030638* | AMEX60DDU001029479 | *AMEX60DDU001029479* |
| AMEX60DDU001001024 | *ALKBH3.L* | AMEX60DD001061 | *AMEX60DD001061* |
| AMEX60DD005167 | *AMEX60DD005167* | AMEX60DDU001025670 | *LOC112543511:25* |
| AMEX60DD034235 | *AMEX60DD034235* | AMEX60DDU001000438 | *GALK1* |
| AMEX60DD000878 | *AMEX60DD000878* | AMEX60DDU001021807 | *AMEX60DDU001021807* |
| AMEX60DDU001035416 | *AMEX60DDU001035416* | AMEX60DDU001022833 | *AMEX60DDU001022833* |
| AMEX60DDU001007283 | *AMEX60DDU001007283* | AMEX60DDU001026828 | *AMEX60DDU001026828* |
| AMEX60DDU001039255 | *AMEX60DDU001039255* | AMEX60DD031363 | *RNF213:18* |
| AMEX60DD045832 | *LOC101935553* | AMEX60DDU001020286 | *AMEX60DDU001020286* |
| AMEX60DD036331 | *LIAS* | AMEX60DDU001012529 | *AMEX60DDU001012529* |
| AMEX60DD011241 | *AMEX60DD011241* | AMEX60DDU001014181 | *AMEX60DDU001014181* |
| AMEX60DD047789 | *SHOX* | AMEX60DDU001033176 | *AMEX60DDU001033176* |
| AMEX60DD003175 | *SST* | AMEX60DDU001018078 | *AMEX60DDU001018078* |
| AMEX60DD007977 | *LEP* | AMEX60DD051774 | *AMEX60DD051774* |
| AMEX60DD010100 | *KRT19* | AMEX60DDU001019802 | *AMEX60DDU001019802* |
| AMEX60DD053674 | *ZBTB16* | AMEX60DDU001032971 | *AMEX60DDU001032971* |
| AMEX60DD028126 | *ZMYND10* | AMEX60DDU001023577 | *AMEX60DDU001023577* |
| AMEX60DD000559 | *CCDC92* | AMEX60DDU001028341 | *AMEX60DDU001028341* |
| AMEX60DD001828 | *SHOX2* | AMEX60DD029826 | *KII* |
| AMEX60DD045037 | *MAB21L2* | AMEX60DD010128 | *KRT17:4* |
| AMEX60DD015986 | *KIRREL1:1* | AMEX60DD008613 | *TNNI1* |
| AMEX60DD005188 | *ALX4* | AMEX60DD046920 | *CST14B.1* |
| AMEX60DD046850 | *TBX15* | AMEX60DD021001 | *TIMP3* |
| AMEX60DD028124 | *HBE1:1* | AMEX60DD029825 | *KII:2* |
| AMEX60DD048589 | *LOC109141055:1* | AMEX60DD021955 | *AMEX60DD021955* |
| AMEX60DD021537 | *HBZ* | AMEX60DD016118 | *LOC108696921:2* |
| AMEX60DD018915 | *AMEX60DD018915* | AMEX60DD054004 | *AMEX60DD054004* |
| AMEX60DD021534 | *N/A:267* | AMEX60DD048317 | *EPHA3* |
| AMEX60DD024041 | *AMEX60DD024041* | AMEX60DD017969 | *AMEX60DD017969* |
| AMEX60DD021531 | *HB-AM* | AMEX60DD018614 | *N/A:143* |
| AMEX60DD030069 | *HAND1* | AMEX60DD053733 | *LOC115074559:2* |
| AMEX60DD003537 | *AMEX60DD003537* | AMEX60DD055613 | *EVX2* |
| AMEX60DDU001000391 | *AMEX60DDU001000391* | AMEX60DD000442 | *LOC107385925:11* |
| AMEX60DD021527 | *HBG1* | AMEX60DD021956 | *EVX1* |
| AMEX60DD026264 | *HBD* | AMEX60DDU001012569 | *AMEX60DDU001012569* |
| AMEX60DD028125 | *HBG2:2* | AMEX60DD038381 | *FOXF2* |
| AMEX60DD026267 | *GLNC1* | AMEX60DD054519 | *NOS2:1* |
| AMEX60DD032898 | *RHAG* | AMEX60DD043673 | *LOC112977635* |
| AMEX60DD042814 | *AMEX60DD042814* | AMEX60DDU001026793 | *AMEX60DDU001026793* |
| AMEX60DD046442 | *AMEX60DD046442* | AMEX60DD022858 | *TMLHE* |
| AMEX60DDU001009716 | *AMEX60DDU001009716* | AMEX60DD012191 | *COCH* |
| AMEX60DD018448 | *PRRX1* | AMEX60DD051976 | *AMEX60DD051976* |
| AMEX60DD025537 | *ALAS2* | AMEX60DD020766 | *MSLN:1* |
| AMEX60DD044522 | *LOC108706699* | AMEX60DD020628 | *UMOD:4* |
| AMEX60DD042402 | *ENC1:1* | AMEX60DDU001010337 | *AMEX60DDU001010337* |
| AMEX60DD049212 | *MAB21L1* | AMEX60DD026773 | *C1S:1* |
| AMEX60DDU001033447 | *AMEX60DDU001033447* | AMEX60DD016113 | *GSONMT00075035001:3* |
| AMEX60DD017008 | *AMEX60DD017008* | AMEX60DD038591 | *VSTM2A* |
| AMEX60DDU001002248 | *CCN1:1* | AMEX60DD049364 | *GJB6:1* |
| AMEX60DD005267 | *CHRM4:1* | AMEX60DDU001005594 | *AMEX60DDU001005594* |
| AMEX60DD051507 | *ALPL* | AMEX60DD031364 | *RNF213:9* |
| AMEX60DD018914 | *ADGRL2* | AMEX60DDU001022902 | *MYH13:2* |
| AMEX60DD037643 | *DACH2* | AMEX60DD026556 | *LOC115078387* |
| AMEX60DD036126 | *TGFB2:1* | AMEX60DD021953 | *HOXA13* |
| AMEX60DD055610 | *HOXD10* | AMEX60DD032523 | *WFDC8* |
| AMEX60DD016542 | *NTN1* | AMEX60DDU001015461 | *AMEX60DDU001015461* |
| AMEX60DD011856 | *SIX1* | AMEX60DD016117 | *GSONMT00055939001:20* |
| AMEX60DD019011 | *CCN1:3* | AMEX60DD039603 | *DSG4:1* |
| AMEX60DD015718 | *CRABP2* | AMEX60DDU001001010 | *AMEX60DDU001001010* |
| AMEX60DD052103 | *AMEX60DD052103* | AMEX60DDU001036789 | *LOC115460730:29* |
| AMEX60DD043155 | *MAMDC2:1* | AMEX60DD029816 | *KRT6A* |
| AMEX60DD022790 | *AMEX60DD022790* | AMEX60DD035815 | *FLRT3* |
| AMEX60DD053152 | *TCF7L2:1* | AMEX60DD032871 | *TFAP2B* |
| AMEX60DDU001019143 | *UFSP2:2* | AMEX60DD012252 | *PAX9* |
| AMEX60DD036020 | *MRPS10* | AMEX60DD018225 | *TNN* |
| AMEX60DD034258 | *CCN2:1* | AMEX60DD049694 | *TENM4* |
| AMEX60DD010576 | *LOC115082302:1* | AMEX60DDU001012072 | *AMEX60DDU001012072* |
| AMEX60DD027737 | *IL17B* | AMEX60DD054520 | *NOS2* |
| AMEX60DD022038 | *CCK* | AMEX60DD010094 | *KRT17:2* |
| AMEX60DD010124 | *KRT12:8* | AMEX60DDU001008861 | *ENV:166* |
| AMEX60DD018616 | *RRBP1:2* | AMEX60DD009557 | *TGM1* |
| AMEX60DDU001011066 | *LOC100130764:43* | AMEX60DD055433 | *LOC115081983* |
| AMEX60DD029451 | *ENDOU:1* | AMEX60DD007033 | *FAM180A* |
| AMEX60DD003483 | *NR2F2* | AMEX60DDU001037591 | *AMEX60DDU001037591* |
| AMEX60DD033617 | *LOC114552212:4* | AMEX60DDU001021749 | *AMEX60DDU001021749* |
| AMEX60DDU001010204 | *LOC115085683* | AMEX60DD029166 | *EMP1* |
| AMEX60DD046276 | *CYP26B1* | AMEX60DD010547 | *AMEX60DD010547* |
| AMEX60DD010875 | *TAP1* | AMEX60DD044914 | *NDNF:1* |
| AMEX60DD018362 | *FMO3* | AMEX60DD031416 | *LGALS3BP* |
| AMEX60DDU001003830 | *E1301_TTI023775:32* | AMEX60DD017703 | *ABHD8* |
| AMEX60DD017446 | *AMEX60DD017446* | AMEX60DD010885 | *AMEX60DD010885* |
| AMEX60DD027485 | *LOC115098183* | AMEX60DDU001003857 | *PARPI_0025344:3* |
| AMEX60DDU001034982 | *LOC112545447:19* | AMEX60DD055612 | *HOXD13* |
| AMEX60DD029959 | *PRPH:2* | AMEX60DDU001042227 | *KRT12:6* |
| AMEX60DD029822 | *KRT5:2* | AMEX60DD024290 | *CHGA* |
| AMEX60DD029384 | *LPO* | AMEX60DD012146 | *Meis* |

**Supporting Information Table 2.**

Primers used for Plasmid construction.

| Primer name | Sequence of the cloning primer (5’-3’) |
| --- | --- |
| tBid-F1 | CGTGGAGGAGAATCCCGGCCCTGGGAATCAATGTAGCAGAATAAGCTATCAC |
| tBid-R1 | GCCTGCACCTGAGGAGTGGATCCTCATTCATCGCCCTGTCTTGTTAAACG |
| Cherry-T2A-F | CTCATCATTTTGGCAAAGAATTATTCCGCTAGCCGCCACCATGGTGAGCAAGGGC |
| Cherry-T2A-R | TTATTCTGCTACATTGATTCCCAGGGCCGGGATTCTCCTCCACGTCAC |
| tBid-F2 | TATAGATCTAAGCTTCTGCAGCCCGGGGCCGCCATGGGGAATCAATGTAGCAGAATAAG |
| tBid-R2 | GTGAATTAATCGATTCATTCATCGCCCTGTCTTGTTAAAC |

**Supporting Information Table 3.**

Primers used for mRNA probe synthesis.

| Primer name | Sequence of the cloning primer (5’-3’) |
| --- | --- |
| Hoxa9-F | TCATATTTTTTCCTGGAGGTCCCGCT |
| T7-Hoxa9-R | TTGAAATTAATACGACTCACTATAGGGTCACTCGTCCTTAGGCCGGTCC |
| Hoxa11-F | TCCTCTTCCGGCAACAACGAGGAGAA |
| T7-Hoxa11-R | TTGAAATTAATACGACTCACTATAGGGAACACATATGTGCATTTAGCCATCGAC |
| Hoxa13-F | TGTTTCCAGTCTGTGGGGCTTT |
| T7-Hoxa13-R | TTGAAATTAATACGACTCACTATAGGGTGACCTTCTTCTCTTTGACCCTCC |
